# The Effect of Proximal Cortical Screw Length of Volar Locking Plates on Clinical Outcomes in Distal Radius Fractures

**DOI:** 10.7759/cureus.81823

**Published:** 2025-04-07

**Authors:** Mahmut Tuncez, Tugrul Bulut, Yilmaz Onder, Abdulhakim Ceyhan

**Affiliations:** 1 Department of Orthopedics and Traumatology, Izmir Katip Celebi University Ataturk Training and Research Hospital, Izmir, TUR; 2 Department of Orthopedics and Traumatology, Katip Celebi University, Izmir, TUR

**Keywords:** dorsal cortex protrusion, radius fracture, screw prominence, volar locking plate, wrist

## Abstract

Objective

The aim of this study is to examine whether the length of the proximal diaphyseal screws of volar locking plates used in distal radius fractures affects the clinical outcomes.

Material and methods

The study included patients who were over 18 years of age and underwent volar locking plate application due to distal radius fracture with a minimum follow-up period of 12 months. Demographic data, Quick Disabilities of the Arm, Shoulder, and Hand (QDASH), grip strength, range of motion, and extensor tendon irritations were evaluated in patients. The prominence of the proximal cortical screws of the plates from the dorsal cortex was measured (in mm) on the lateral radiographs of the patients.

Results

The median follow-up time of the 73 patients in our study was 17 (14-26) months. The median age of the patients was 51 (44-58) years. Extensor tenosynovitis was detected in 22 patients due to proximal screw length. Proximal screw prominence over 1.2 mm was found to be statistically significant for extensor synovitis (p<0.05). Range of motion, radiological measurements, grip strength, QDASH, and other demographic data had no association with extensor tenosynovitis.

Conclusion

In the surgical treatment of distal radius fractures, dorsal cortex protrusion of proximal cortical screws of volar locking plates more than 1.2 mm plays a role in postoperative patient dissatisfaction.

## Introduction

Distal radius fractures (DRFs) are among the most common fractures seen in adults [1]. Therefore, there are many options for their treatment. Although closed reduction is the most common, there are also DRFs that require surgery [2-3]. The main indications for surgery are unstable DRF and comminuted intra-articular fractures, especially in young people. Surgical treatment has been shown to have a positive effect on functional outcomes in short-term follow-up [4]. Volar locking plates (VLPs) have become the dominant method in the surgical treatment of DRFs over the last 20 years [3-5]. In addition to being biomechanically stable, VLPs present less risk of tendon irritation compared to dorsal plates. However, extensor tendon complications have also been reported in patients undergoing treatment with VLP [6-8]. This situation/complication negatively affects clinical outcomes despite the anatomical reduction achieved with successful surgery. Therefore, it may cause patient dissatisfaction and overshadow the surgeon's success.

Extensor tendon complications in patients with DRF treated with VLP are due to various causes. One of these is extensor pollicis longus tendon ruptures, which may occur as a result of the injury itself [9]. Another may be associated with iatrogenic injury to the extensor tendons, prior perforation of the dorsal cortex, or screw prominence [10]. There are not enough clinical studies examining the clinical outcomes related to the number of proximal cortical screws and proximal cortical screw length (PSL) in VLPs applied to DRFs [11].

Our hypothesis is that clinical outcomes will be negatively affected due to extensor tendon irritation that will occur as the length of the proximal cortical screws protruding the dorsal cortex increases in VLPs applied for DRFs.

The aim of this study is to evaluate the effect of the length of screws applied to the diaphysis of VLPs used in DRFs on clinical outcomes.

## Materials and methods

After institutional review board approval, a retrospective review was conducted to identify patients who had undergone volar locked plating of a DRF between January 2020 and January 2024 at our institution (IRB#97/2024). This study was conducted in accordance with the principles of the Declaration of Helsinki.

Patient inclusion

**Figure 1:**
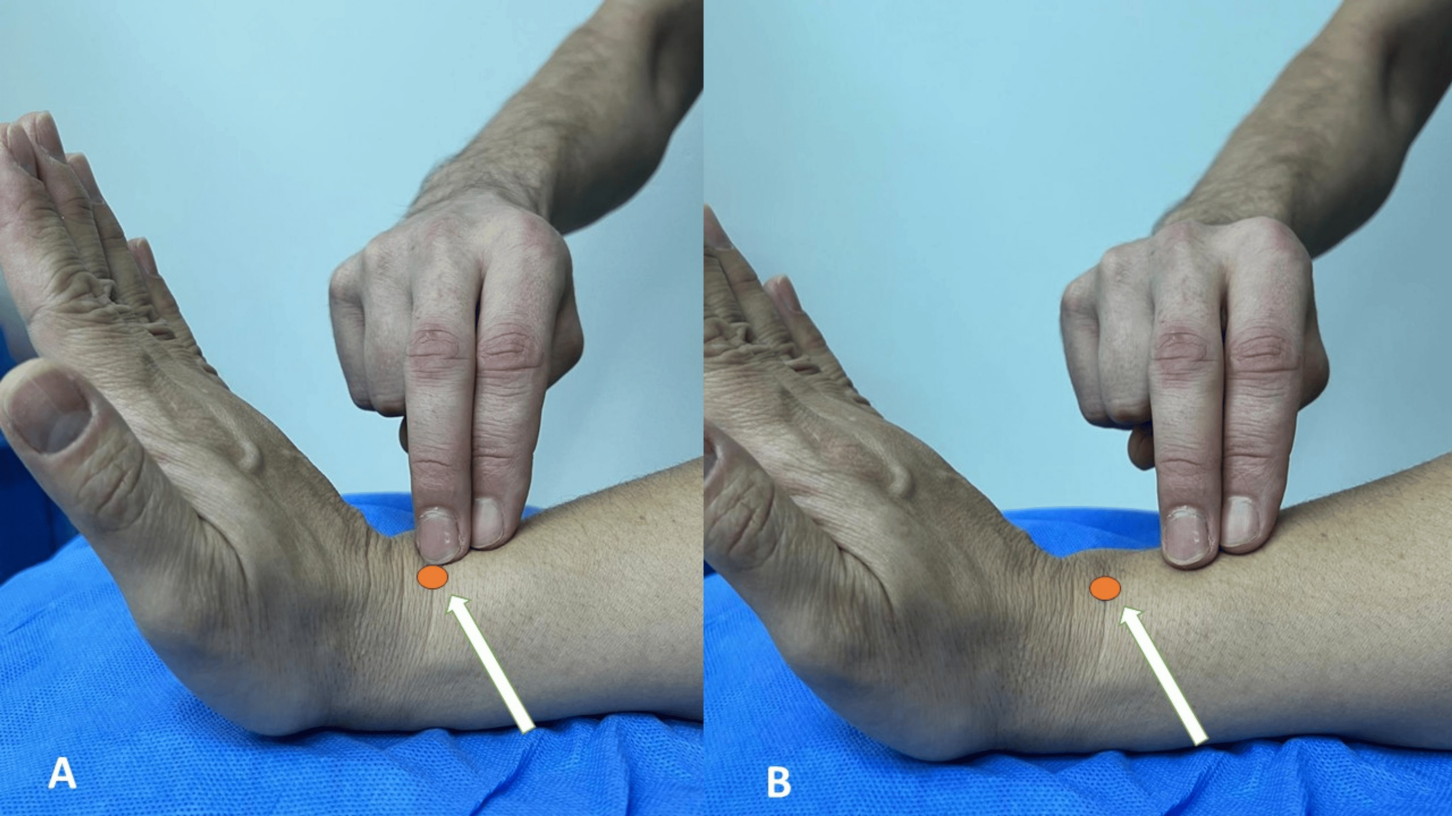
(A) Palpation method on Lister's tubercle (brown circle, white arrow) with the wrist and hand in extension for distal screw tenosynovitis. (B) Palpation method proximal to Lister's tubercle (brown circle, white arrow) with the wrist and hand in extension for proximal screw tenosynovitis.

Patients over the age of 18 who underwent VLP and patients with a minimum follow-up period of 12 months were included in the study. Patients with concomitant injuries in the same extremity, those who underwent non-VLP surgical treatment such as external fixation or percutaneous pinning, those with open fractures, those who underwent plate or screw removal due to the distal screw being long or intra-articular, and those who did not come for their final follow-up were excluded from the study. In addition, patients with extensor tenderness caused by distal screws (tenderness with active hand extension over Lister’s tubercle and palpation in extensor compartments) (Figure 1A) and patients whose plates were removed because the distal screws were intra-articular were excluded from the study.

Evaluation of patients

For clinical outcomes, the Quick Disabilities of the Arm, Shoulder, and Hand (QDASH), grip strength, range of motion, extensor tenosynovitis, extensor tendon ruptures, complications, and reoperations were evaluated at the final follow-up.. Range-of-motion measurements were measured as the difference between the patient's healthy side and the fractured side.

Grip strength was measured on both the fractured and healthy sides using a Jamar hand dynamometer (Asimow Engineering, Los Angeles, CA, USA), and the difference between the two was recorded. QDASH, another method used in functional assessment, is a questionnaire that allows us to evaluate the functional capacity of the upper extremity by questioning the ability to perform some daily physical and social activities and the severity of symptoms such as pain.

At the last follow-up of the patients, extensor tenosynovitis was noted by palpation and anamnesis (Figure 1B). To distinguish whether extensor tenosynovitis was caused by distal or proximal screws, patients with pain during extension were divided into those with tenderness on palpation around Lister's tubercle, those with tenderness proximal to Lister's tubercle (radial shaft), and those with pain at both points. Patients with dissatisfaction around Lister's tubercle and at both points were excluded from the study (26 patients).

**Figure 2:**
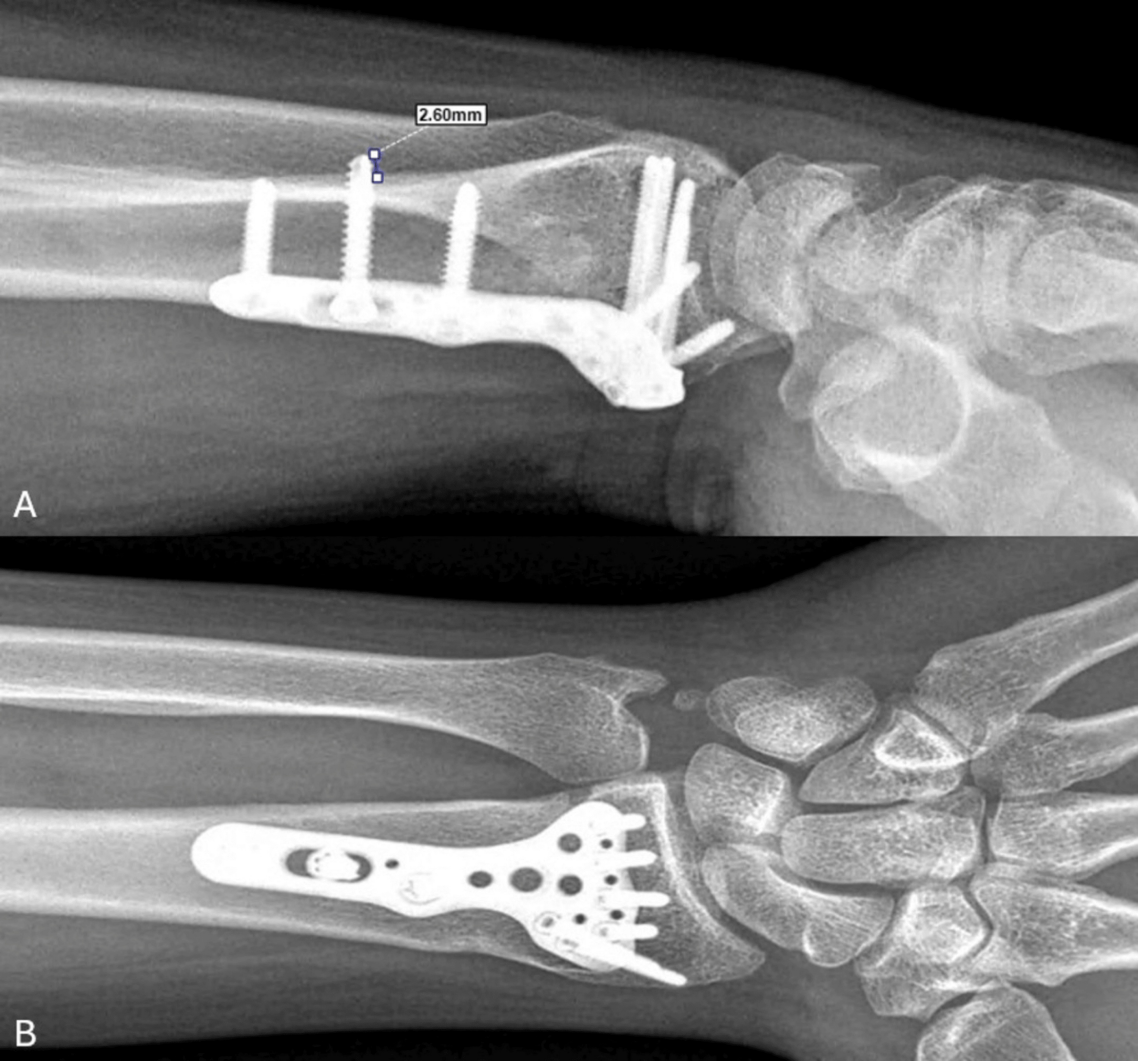
Postoperative 15-month wrist X-ray of a 41-year-old male patient. (A) Measurement of the dorsal radius cortex with the most prominent screw on a lateral direct radiograph of a healed distal radius fracture treated with a volar plate. (B) Posteroanterior wrist X-ray of the same patient.

Posteroanterior and lateral digital radiographs of the patients obtained from picture archiving and communication systems (PACS) were used for radiologic measurements. Radiologically, radial inclination, volar tilt, radial height, and ulnar variance were measured on the patients' preoperative and final X-rays. Dorsal cortex prominence was measured from the most prominent direct radiograph of the patients postoperatively. The most prominent proximal cortical screw was identified. Top of the screw was made and measured from the outer dorsal cortex of the radial shaft to the tip of the screw (Figure 2). All radiological measurements were performed together with consensus by a team including two orthopedic surgeons. All measurements were done with the digital angle and distance measurement tools in the PACS.

Surgical treatment

All patients were prepared in the supine position with infraclavicular block anesthesia. The fracture line was reached with a Colar Henry incision using a pneumatic tourniquet of 250 mmHg/90 min. After reduction of the fracture, it was fixed with a VLP. Distal locking screws were performed as a single cortex. A volar distal radius plate with 2.7-mm distal screw and 3.5-mm proximal screw (Acu-Loc, Acumed, Hillsboro, OR, USA) was used for all patients. Proximal screws were used as a double cortex. After bleeding control, the skin incision was closed and a short arm splint was applied. Active finger range of movements was started immediately, and after two weeks, the short arm splint was terminated and wrist movements were started. Strengthening was delayed for at least six weeks until fracture union.

Statistical analysis

Analysis of the data obtained in the study was conducted using IBM SPSS Version 26 (IBM Corp., Armonk, NY, USA). The suitability of continuous variables was evaluated with graphical research (Q-Q plot), normality tests (Shapiro-Wilk), and sample size. It was found that they did not meet normal distribution conditions. Continuous variables were presented as median and IQR (25-75). Comparisons of independent groups were done using the 'Mann-Whitney U test'. Categorical independent variables were presented as frequencies and percentages with cross-tabulations. Their distributions were compared using the chi-square test methods. The receiver operating characteristic (ROC) analysis was performed for PSL, and the most appropriate cut-off value was determined according to the Youden index. Dichotomous variables were created according to these cut-off values, and odds ratios were calculated. In all statistical comparison tests, the margin of type 1 error was determined as α 0.05, and two-tailed tests were performed.

## Results

**Figure 3:**
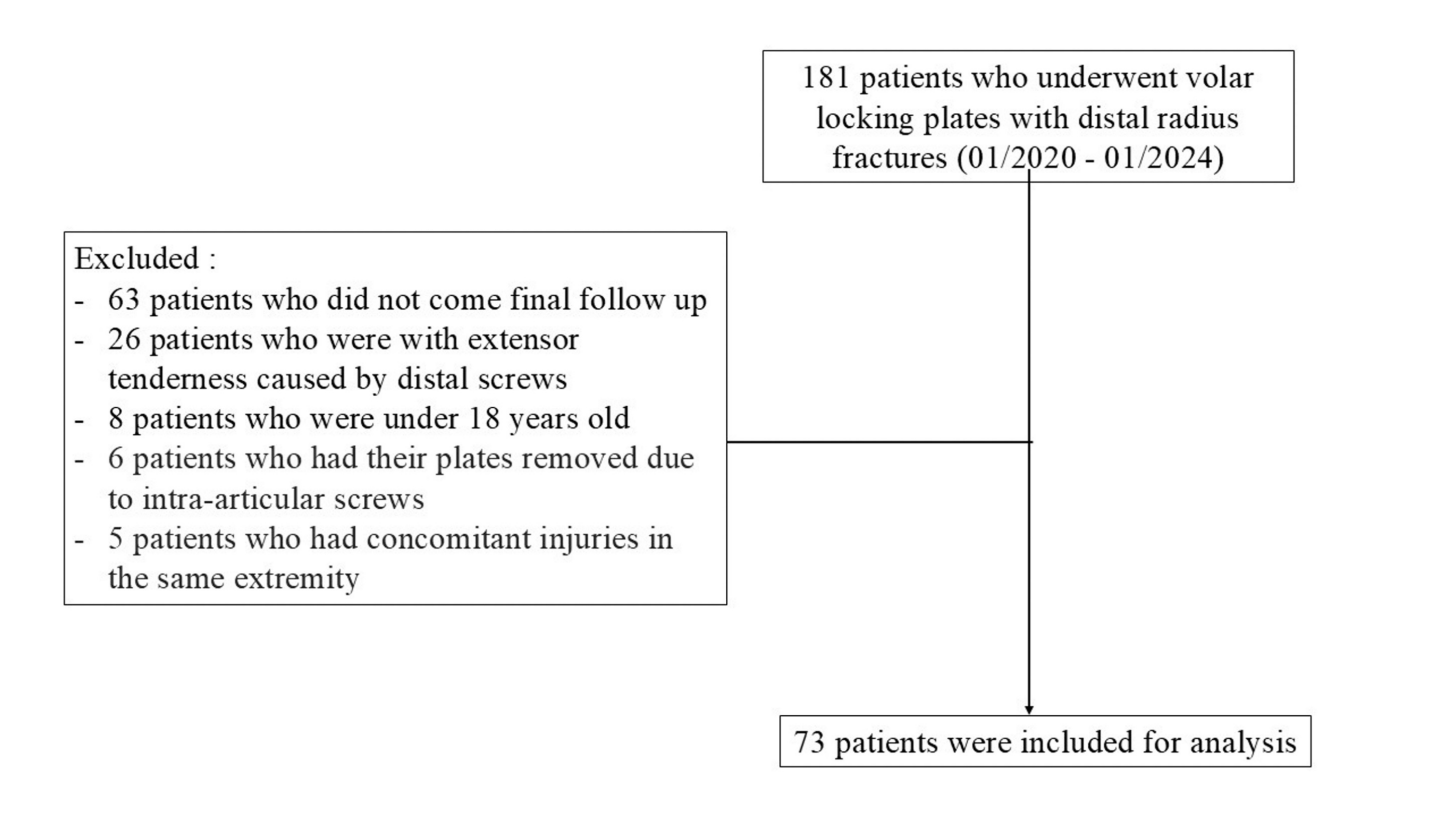
Flow chart of the distribution of patients.

Of the 181 patients who underwent volar plates, 73 who met the inclusion criteria were included in the study (Figure 3). Majority of the patients who underwent the plating operation were men (n = 42 [58%] men and n = 31 [42%] women). The median age was 51 years (range: 19 to 76 years), and the median follow-up period was 17 months (range: 12 to 48 months) (Table 1).

**Table 1:** Demographic data of patients and their relationship with extensor tenosynovitis Test statistics: chi-square (χ²) and 'Mann-Whitney U test', depending on the variable type. *A p-value of <0.05 was considered significant. QDASH, Quick Disabilities of the Arm, Shoulder, and Hand

	Extensor sensitivity			P-value
	Total (73)	Yes (22)	No (51)	
	Median (IQR) / N (%)	Median (IQR) / N (%)	Median (IQR) / N (%)	
Age	51 (44-58)	51 (42-57)	51 (44-59)	0.678
Sex				
Male	42 (58%)	13 (59%)	29 (57%)	1.000
Female	31 (42%)	9 (41%)	22 (43%)	
Fracture side				
Right	45 (62%)	13 (59%)	32 (63%)	0.974
Left	28 (38%)	9 (41%)	19 (37%)	
Fracture on the dominant side				
Yes	44 (60%)	10 (45%)	34 (67%)	0.150
No	29 (40%)	12 (55%)	17 (33%)	
Follow-up duration	17 (14-26)	15 (13-17)	20 (15-32)	0.110
Grip strength (non-fractured side-fractured side) (kilogram)	18 (12-26)	17 (12-20)	18 (14-26)	0.316
QDASH	9.1 (2.3-20)	9.1 (2.3-25)	6.8 (2.3-18.2)	0.620
Volar tilt preoperative	-15 (-20 to -3)	-14 (-19 to -10)	-15 (-23 to 6)	0.643
Volar tilt postoperative	10 (6-10)	10 (4-12)	10 (6-10)	0.990
Radial inclination preoperative	18 (14-20)	16 (14-20)	18 (14-20)	0.947
Radial inclination postoperative	20 (18-22)	21 (16-22)	20 (18-21)	0.255
Radial height preoperative	7 (3-10)	8 (3-11)	7 (4-10)	0.708
Radial height postoperative	10 (8-11)	10 (8-12)	10 (9-11)	0.841
Number of proximal screws				
2 screws	17 (23%)	3 (14%)	14 (27%)	0.327
3-4 screws	56 (77%)	19 (86%)	37 (73%)	
Proximal screw length (mm)	1 (0.6;2)	2.1 (1.5;3)	0.8 (0.5;1.5)	<0.001*
Proximal screw length				
>1.2 mm	32 (44%)	18 (82%)	14 (27%)	<0.001*
≤1.2 mm	41 (56%)	4 (18%)	37 (73%)	

Twenty-two (30%) patients were found to have clinically significant extensor tendon irritation, with no patient with an extensor tendon rupture. There was no statistical difference between patients with and without extensor tenosynovitis in terms of QDASH, grip strength, and range of motion.

**Figure 4:**
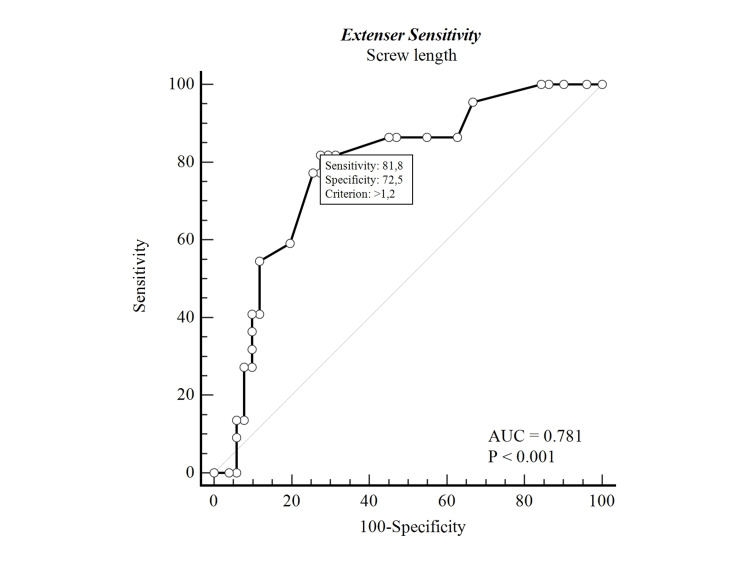
ROC analysis for extensor sensitivity and proximal screw prominence. AUC, area under the curve ROC, receiver operating characteristic

When the proximal screw number was examined between two and more than two patients, no statistical difference was found in the clinical and radiological results. However, the dorsal cortex protrusion of PSL was found to be statistically significantly higher in patients with extensor tenosynovitis (Table 1). The ROC curve analysis was performed, and extensor tenosynovitis was found to be statistically significant in patients with the dorsal cortex protrusion of PSL of over 1.2 mm (Figure 4).

## Discussion

Extensor tendon irritation is more common than thought in DRFs treated with volar plating. When we look at the literature, we usually talk about extensor irritation without distinguishing between the distal screws or the proximal screws of the volar plate [12-14]. In our study, we examined the status of the screws by focusing on the radius shaft part and showed that if the dorsal cortex protrusion of PSL is longer than 1.2 mm, it can cause significant extensor tenosynovitis. This could be due to the focus on placing distal screws rather than proximal cortical screws during the operation and the fluoroscopy not being in appropriate supination-pronation.

Wall et al. showed that 75% of unicortical screws provide similar biomechanical stiffness to bicortical screws [15]. White et al. found that dorsal cortex prominence less than or greater than 2 mm was positively correlated with tendinopathy and tendon rupture [16]. Hong et al. also found similar results [17]. On the other hand, Pulos et al. found in their study that proximal screw prominence was not clinically significant [11]. However, Pulos et al. evaluated patients who underwent VLP for DRFs and those who underwent implant removal without considering the reason for the implant removal [11]. In our study, we evaluated PSL in a more homogeneous group by excluding patients with extensor tendon irritation due to distal screws. In our study, where we evaluated only proximal tendon tenderness, we found that patients with the dorsal cortex protrusion of PSL of 1.2 mm and above had significantly worse clinical outcomes. We believe that this complication can be prevented by applying proximal cortical screws as a single cortex or by keeping the protrusion amount less than 1.2 mm.

Eng et al. reported in their study that using 10 degrees of increasing supination-pronation while applying proximal screws will reduce screw prominence and complications [18]. Since our study is retrospective in nature, we could not use a direct radiograph obtained with a standard supination-pronation. However, we preferred the one with the most prominence of PSL among the direct radiographs obtained during follow-up. We believe that the degree of supination-pronation is important when obtaining intraoperative fluoroscopy. However, this may increase the amount of radiation exposure.

There are a few limitations of the present study. The most important limitations of this study are the relatively small number of patients and the fact that it is conducted in a single center. Another limitation of this study is that due to its retrospective design, the standard pronation or supination angle could not be determined on postoperative direct radiographs. For this reason, we examined all postoperative radiographs of the patients and made measurements on the most appropriate X-ray image. Another limitation of the study is that extensor tendon irritation was evaluated by recording the pain on palpation, which subjective data.

## Conclusions

VLPs are widely used in the surgical treatment of DRFs; however, dorsal cortex protrusion of proximal cortical screws of VLPs of more than 1.2 mm plays a role in patient dissatisfaction. If the surgeon wants to achieve successful and satisfactory outcomes with VLP application in DRFs, they should pay attention to the proximal cortical screws as much as they pay attention to the quality of reduction and the position and length of the distal screws. Because, the proximal cortical screws are long, they may cause patient dissatisfaction due to extensor tendon irritation despite a successful surgery.

## References

[1] Court-Brown CM, Caesar B (2006). Epidemiology of adult fractures: a review. Injury.

[2] Huetteman HE, Shauver MJ, Malay S, Chung TT, Chung KC (2019). Variation in the treatment of distal radius fractures in the United States: 2010 to 2015. Plast Reconstr Surg.

[3] Mellstrand-Navarro C, Pettersson HJ, Tornqvist H, Ponzer S (2014). The operative treatment of fractures of the distal radius is increasing: results from a nationwide Swedish study. Bone Joint J.

[4] Orbay JL, Fernandez DL (2002). Volar fixation for dorsally displaced fractures of the distal radius: a preliminary report. J Hand Surg Am.

[5] Smith DW, Henry MH (2005). Volar fixed-angle plating of the distal radius. J Am Acad Orthop Surg.

[6] Arora R, Lutz M, Hennerbichler A, Krappinger D, Espen D, Gabl M (2007). Complications following internal fixation of unstable distal radius fracture with a palmar locking-plate. J Orthop Trauma.

[7] Azzi AJ, Aldekhayel S, Boehm KS, Zadeh T (2017). Tendon rupture and tenosynovitis following internal fixation of distal radius fractures: a systematic review. Plast Reconstr Surg.

[8] Berglund LM, Messer TM (2009). Complications of volar plate fixation for managing distal radius fractures. J Am Acad Orthop Surg.

[9] Engkvist O, Lundborg G (1979). Rupture of the extensor pollicis longus tendon after fracture of the lower end of the radius--a clinical and microangiographic study. Hand.

[10] Al-Rashid M, Theivendran K, Craigen MA (2006). Delayed ruptures of the extensor tendon secondary to the use of volar locking compression plates for distal radial fractures. J Bone Joint Surg Br.

[11] Pulos N, DeGeorge BR Jr, Shin AY, Rizzo M (2020). The effect of radial shaft dorsal screw prominence in volar locking plate fixation of distal radius fractures. Hand (N Y).

[12] Sügün TS, Karabay N, Gürbüz Y, Ozaksar K, Toros T, Kayalar M (2011). Screw prominences related to palmar locking plating of distal radius. J Hand Surg Eur Vol.

[13] Austin A, Green S, Ahsan S, Roskosky M, Shuler MS (2015). Cadaveric study of appropriate screw length for distal radius stabilization using volar plate fixation. Am J Orthop (Belle Mead NJ).

[14] Ljungquist KL, Agnew SP, Huang JI (2015). Predicting a safe screw length for volar plate fixation of distal radius fractures: lunate depth as a marker for distal radius depth. J Hand Surg Am.

[15] Wall LB, Brodt MD, Silva MJ, Boyer MI, Calfee RP (2012). The effects of screw length on stability of simulated osteoporotic distal radius fractures fixed with volar locking plates. J Hand Surg Am.

[16] White BD, Nydick JA, Karsky D, Williams BD, Hess AV, Stone JD (2012). Incidence and clinical outcomes of tendon rupture following distal radius fracture. J Hand Surg Am.

[17] Hong DY, Kunes JA, Tedesco LJ, Danford NC, Strauch RJ (2023). Anatomic risks with overpenetration of the volar locking plates' proximal holes. J Wrist Surg.

[18] Eng K, Gil S, Page R (2020). Diaphyseal screw prominence in distal radius volar plating. J Wrist Surg.

